# Serotonin as a volume transmission signal in the “simple nervous system” of mollusks: From axonal guidance to behavioral orchestration

**DOI:** 10.3389/fnsyn.2022.1024778

**Published:** 2022-11-08

**Authors:** Elena E. Voronezhskaya

**Affiliations:** Laboratory of Comparative and Developmental Physiology, Koltsov Institute of Developmental Biology, Russian Academy of Sciences, Moscow, Russia

**Keywords:** serotonin, larval apical organ, axonal navigation, developmental tempo, gating mediator, behavior adaptation, central pattern generator (CPG)

## Introduction

The main idea of neurobiology is to understand how the nervous system emerges during development and later organizes the complex behavior of the adult. This process is based on the activity of individual neurons, the formation of neuronal connections, and the release of neurotransmitters and neurohormones by the nerve cells. A small brain weighing one-third of a gram contains about 100 million neurons, a corresponding number of neuronal connections, and hundreds of actively released substances. So if someone were to try to uncover the principles of neuronal organization, they probably would not start with such a complicated system. Although the main interest of researchers is in the field of vertebrates and mammals (including humans), scientists often use simpler systems to understand the general principles of the nervous system development and organization.

More than 200 years of research have shown that such an approach is truly productive. Today, there is no doubt that the structural elements of the brain—whether it is a simple neural network, ganglia, or a complex multilayered structure—are similar. Neurons differentiate according to homologous genetic programs; structural elements of the brain share comparable morphological features such as neurons, axons and synapses, as well as a common biochemical background and molecular signaling pathways. This allows researchers to select the most appropriate model system for their specific task. Mollusks are one of the most popular model systems for neurobiologists because they have a limited number of neurons that arise sequentially in the course of neurogenesis, many neurons can be individually identified, thick axons allow for the labeling of neuron projection areas in the body, and immunochemical markers visualize cells that contain specific neurotransmitters.

In general, the brain of mollusks consists of several ganglia with nerve bodies on the ganglion surface and projecting processes that are organized into an inner ganglionic structure: the neuropil. The axons leaving the ganglia *via* the nerves to the peripheral targets are responsible for motor control of the various organs, while the processes entering the ganglia from the periphery provide the sensory information. The processing of the received information takes place in the ganglia at the level of hierarchical neuronal interactions, and the output rhythmic activity is generated by central pattern generators (CPGs). In addition, numerous peripheral neurons (sometimes condensed into ganglia) organize networks that process information locally; and varicose fibers in the neuropil release neurotransmitters that influence neuronal activity within ganglia. Mollusks demonstrate behaviors that can be analyzed as separate acts often organized as repetitive (rhythmic) patterns (for example, locomotion, swimming, and feeding). Certain behavioral acts (escape and learning) occur in response to external stimuli or their specific combinations. In summary, the entire system looks intuitively logical, making mollusks an attractive model for analyzing the input of a particular neurotransmitter on behavior.

Serotonin (5-hydroxytryptamine, 5-HT) is a biogenic amine with well-known functions as a neurotransmitter and neuromodulator. It is found in the central and peripheral nervous systems of vertebrates and invertebrates and influences various aspects of the organism, from arousal responses, learning, and feeding to aggression and sleep (Müller and Cunningham, 2020). Serotonin represents an evolutionarily ancient and archetypical signaling system (Turlejski, 1996; Azmitia, 2020), it occurs early in animal development, beginning in oocytes, zygotes, and cleaved blastomeres (Buznikov et al., 1964, 2001; Dubé and Amireault, 2007). The serotonergic system, including enzymes for the synthesis and degradation of 5-HT, receptors and coupled G proteins, membrane transporters, is among the first to be expressed in differentiating neurons of the forming nervous system (Schmidt-Rhaesa et al., 2016; Müller and Cunningham, 2020). All components of the serotonergic system demonstrate high plasticity, with both the anatomical organizations and the biochemical machinery changing at different stages of life or under various environmental conditions. All of the above characteristics give serotonin a high potential to serve as a general modulator or integrating molecule at the level of the whole organism. This idea was introduced by Dmitry Sakharov (Sakharov D., 1990; Sakharov D. A., 1990; Kabotyanski and Sakharov, 1991) and later confirmed by numerous studies (summarized by Dyakonova, 2007).

**Figure 1:**
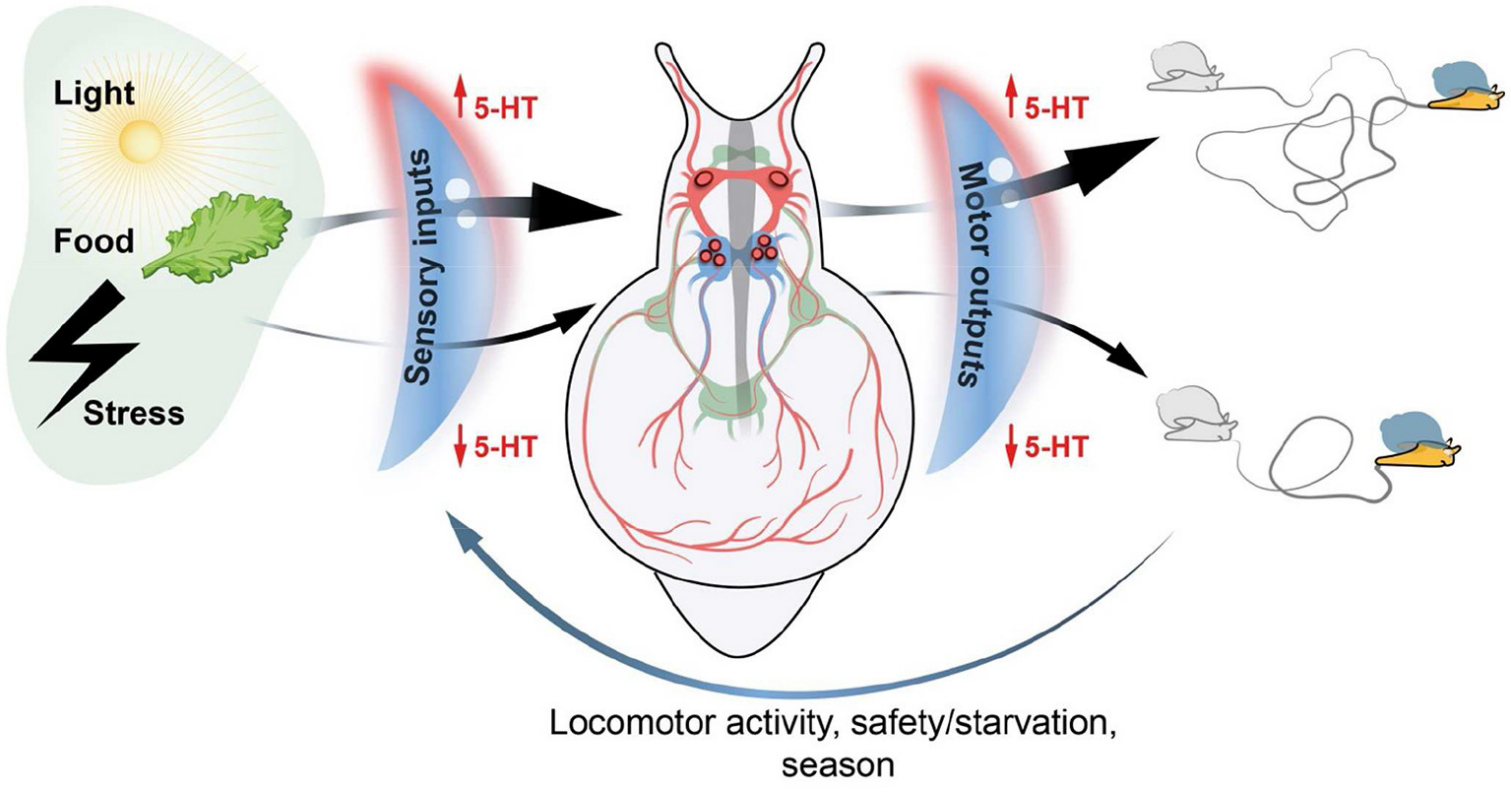
Schematic representation of integrative serotonin action. Serotonin (5-HT) acts as a volume transmission signal tuning both sensory inputs and motor outputs in the nervous system of mollusks. 5-HT has a gating modulatory function for external stimuli of various modalities by amplifying or attenuating the signal generated by sensory neurons. 5-HT also acts as a gating mediator required for certain activities of the central neural circuits (Central Pattern Generators) which generate high or low motor output and the corresponding activity of the animal. A complex of internal and external factors (locomotor activity, satiety/starvation state, and season) in turn influences 5-HT level within the nervous system and in the peripheral local networks. Thus, the serotonin level in local area surrounding certain peripheral and central neurons orchestrates the complex behavior of the animal and enables the organism to adapt current situation.

The purpose of my article is to summarize in a brief text the views and ideas about the actions of serotonin as volume transmission signal during development and its function as integrative factor underlying coordinated behavior that I have accumulated over years of study the mollusks model system. All data are presented according to the Sakharov's paradigm about the integrative and orchestrating role of serotonin. I hope that the views I present will encourage young researchers to look at their findings through the lens of transmitter-driven behavioral regulation (Figure 1).

## Serotonin secreted from apical sensory cells navigate axonal growth in early larvae

When can serotonin be detected in neurons and what is its function during molluscan development? Monoamines not only act as intercellular signals that determine the state of electrical activity, but also play an important role in the establishment and maintenance of neuronal morphology and connectivity. The multifunctional role of serotonin and dopamine has been demonstrated in experiments by the groups of Kater and Goldberg. Their landmark work demonstrates the multifunctional role of these bioactive compounds and proves that they are involved in controlling the expression of behavioral repertoires, regulating neurite outgrowth, and controlling the assembly of functional neuronal circuits (summarized in Kater and Haydon, 1987). During molluscan development, the earliest cells expressing serotonin belong to a provisional larval structure—the apical sensory organ (ASO). The ASO—often referred to as the “larval brain”—is the most prominent neural structure located at the apical pole of the larva (Richter et al., 2010). The varicose processes of serotonin-containing vase-shaped ASO cells organize a compact neuropil near the cell bodies. The morphology of this structure suggests that the region serves as an active serotonin secretion area within the larval body (Kempf et al., 1997; Nezlin and Voronezhskaya, 2017). Simultaneously with the ASO neurons or slightly earlier, the second population of neurons begins to differentiate in the larvae. These cells are positive for antibodies against peptide FMRFamide and are also located in the periphery, but in the posterior part of the larval body. The anteriorly directed processes of these posterior cells follow the curvature of the larval body and grow anteriorly (Voronezhskaya and Ivashkin, 2010; Voronezhskaya and Croll, 2015). Near the ASO neuropil, the processes bend and run to the ventral part of the larvae, usually in the region of the foot rudiment. The morphology, location and trajectory of the early posterior cell processes in different species suggest that they form a scaffold and pioneer the structures of the developing nervous system (Croll and Voronezhskaya, 1996; Voronezhskaya and Elekes, 1996; Nezlin and Voronezhskaya, 2017). To navigate the correct path in the larval body, the growth cones probably use the chemical gradients or signals from the guidepost cells. Our experiments have shown that the serotonin gradient is crucial for the correct growth of pioneer axons. Disruption of the 5-HT gradient by administration of external 5-HT (10^−6^ M) resulted in chaotic sprouting of axons, irreversible malformations in the forming ganglia, and eventually larval death (Nezlin and Voronezhskaya, 2017; Yurchenko et al., 2018). These results confirm the hypothesis that serotonin, produced by ASO neurons and released from the compact ASO neuropil, is involved in the navigation of pioneer axons of early posterior cells.

## Developmental tempo is alternatively regulated by serotonin released from the apical sensory cells at premetamorphic and metamorphic larva stages

The apical cells produce and release serotonin during all larval stages. We have shown that tonic release of 5-HT slightly retards developmental tempo and blocking 5-HT synthesis can accelerate development (Voronezhskaya et al., 2004). Interestingly, serotonin had opposite effects in early (pre-metamorphic) and late (metamorphic) larval stages. While the substance released is the same, the combination of receptors expressed and corresponding G-proteins vary in a stage-dependent manner. As a result, the embryonic developmental tempo slows down in response to serotonin at the pre-metamorphic stage, while it accelerates at the metamorphic stage (Glebov et al., 2014). ASO cells respond to the chemical signals released by adults under conditions such as starvation or overcrowding (Voronezhskaya et al., 2004). These results indicate that parent snails adequately inform their offspring of the adverse environmental conditions to which they will be faced after hatching. Serotonin released by ASO works as volume transmission signal and adaptively modulates development according to the current developmental stage and incoming signals, ensuring better embryo survival and offspring dispersal (Voronezhskaya, 2021).

## Serotonin modulates both sensory inputs and motor outputs according to the physiological state of the animal

There are two main approaches to how neurons organize animal behavior. The first theory is based on the electrical activity of individual cells, their synaptic connections, and the subsequent output of processed signals from cell assemblies to targets. The activity of individual cells is modulated by feedback from the sensory periphery. In such a concept, the chemical nature of individual neurons is not critical, and all activity can occur through electrical synapses. With slight variations, this view represents the neurobiological implementation of classical Pavlovian theory. It has been supported by numerous electrophysiological experiments and applied in the engineering of complex technical devices and modern gadgets (the perfect examples are the Boston Dynamics robots). Despite its incredible success, this concept leaves open the question of the multiplicity of neurotransmitters in the real nervous system (Kandel et al., 1984). In the early 1960s, Dmitry Sakharov begins to advocate the alternative view of behavioral regulation. The core of Sakharov's hypothesis became transmitter-dependent behavioral states. According to this view, individual neurotransmitters (e.g., serotonin, dopamine, enkephalins, etc.) not only transmit the signal between neurons but play an integrative role. In this hypothesis, the neurotransmitter also activates specific neurons. However, the triggered activity of the various cells within the different circuits (or CPGs) is not chaotic, but is released as a coordinated motor output that is expressed in the animal's organized behavior acts (e.g., swimming, hunting, food intake and acquisition, and escape locomotion). This type of coordinated organization can be referred to as “orchestration”. The neuroactive substances in the CNS of mollusks are released into the neuropil region and blood and reach the neuronal synapses. The activity of individual neurons in CNS can be modulated by a rich and dynamic chemical microenvironment that reflects the physiological state of the organism (Sakharov, 1974, 2012; Sakharov D., 1990). Changes in the “mediator cocktail” surrounding CPG cells account for the often observed flexibility of many CPG circuits, where a single neuronal circuit can produce a variety of different outputs (Croll et al., 1985; Benjamin, 2012; Sakharov, 2012; Ito et al., 2013). The simple nervous system of mollusks and other invertebrates represents a unique system in which the concept of transmitter-driven behavior has been experimentally tested and studied at the level of individual neurons and neuromediators (Sakharov, 1994, 2012; Libersat and Pflueger, 2004; Dyakonova, 2007). Sakharov's ideas about the non-synaptic organization of coordinated behavior were truly revolutionary. They were accepted by the scientific community only after decades of persistent experimental research. Today, two main modes of intercellular communication in the central nervous system are equally acknowledged: wiring transmission (WT) and volume transmission (VT) (Agnati et al., 2010; Taber and Hurley, 2014). Studies of complex brains add variations in the forms of volume transmission (Fuxe et al., 2013) that play a role in neurogenerative and psychiatric disorders in high organisms. Thus, research on models with simple nervous systems (such as mollusks) contributes greatly to our understanding of the general principles of nervous system functioning.

The important research application for simple nervous systems is that the same neuronal circuit underlying a particular behavior can be analyzed using both wiring and volume transmission approaches. A nice example is the feeding system of gastropod *Lymnaea*. The activity of the network that generates motor patterns can be modulated either by firing of identified neurons (Brierley et al., 1997; Benjamin, 2012) or by releasing specific neurotransmitters (Elliott and Vehovszky, 2000). In either case, the final activity of the buccal muscles responds in a coordinated manner. Serotonin has been shown to be one of the general modulatory substances in mollusks. Moreover, serotonin is involved in a specific type of modulation—the “gating” function. It has been demonstrated that a sufficient level of 5-HT is required to enable neurons in the CPG to drive a feeding rhythm (Yeoman et al., 1994). On the other hand, serotonin level modulates the perception of chemical, visual, and tactile stimuli (Dyakonova and Sakharov, 1995; Nezlin and Voronezhskaya, 1997; Zhukov et al., 2006). Serotonin levels in the CNS of mollusks are very flexible and reflect the state of satiety or hunger (Hernádi et al., 2004), previous locomotor activity (Aonuma et al., 2020), and season (Ivashkin et al., 2015). In general, serotonin levels depend on the previous behavioral context, i.e., a complex of internal and external factors (Ito et al., 2013; Dyakonova, 2014; Bacqué-Cazenave et al., 2020). Thus, we can say that serotonin is a gating mediator for both incoming sensory inputs and motor outputs. In turn, the implementation of various motor programs and contextual behaviors influences the level of serotonin in the CNS. These repeated cycles precisely coordinate and tune the animal's physiological state and behavior to current environmental cues, ensuring the individual's adaptation and survival (Figure 1).

## Author contributions

The author confirms sole responsibility for the article conception and design, data interpretation, and manuscript preparation.

## Funding

The work with serotonin effects on development was supported by the Russian Science Foundation Grant No. 22-14-00375. The original research with 5-HT receptors was conducted under IDB RAS RP # 0008-2021-0020 using Core Centrum facility, and the effects of serotonin on development were supported by RSF # 22-14-00375.

## Dedication

The author would like to dedicate this work to my scientific mentor Dmitry Sakharov, whose ideas about the organization of living systems have inspired me throughout my research life.

## Conflict of interest

The author declares that the research was conducted in the absence of any commercial or financial relationships that could be construed as a potential conflict of interest.

## Publisher's note

All claims expressed in this article are solely those of the authors and do not necessarily represent those of their affiliated organizations, or those of the publisher, the editors and the reviewers. Any product that may be evaluated in this article, or claim that may be made by its manufacturer, is not guaranteed or endorsed by the publisher.
